# PMSTD-Net: A Neural Prediction Network for Perceiving Multi-Scale Spatiotemporal Dynamics

**DOI:** 10.3390/s24144467

**Published:** 2024-07-10

**Authors:** Feng Gao, Sen Li, Yuankang Ye, Chang Liu

**Affiliations:** 1 College of Intelligent Systems Science and Engineering, Harbin Engineering University, Harbin 150001, China; 2 Qingdao Innovation and Development Center, Harbin Engineering University, Qingdao 266400, China; 3 Qingdao Hatran Ocean Intelligence Technology Co., Ltd., Qingdao 266400, China

**Keywords:** dynamic change, multi-scale, spatiotemporal prediction, sensor data

## Abstract

With the continuous advancement of sensing technology, applying large amounts of sensor data to practical prediction processes using artificial intelligence methods has become a developmental direction. In sensing images and remote sensing meteorological data, the dynamic changes in the prediction targets relative to their background information often exhibit more significant dynamic characteristics. Previous prediction methods did not specifically analyze and study the dynamic change information of prediction targets at spatiotemporal multi-scale. Therefore, this paper proposes a neural prediction network based on perceptual multi-scale spatiotemporal dynamic changes (PMSTD-Net). By designing Multi-Scale Space Motion Change Attention Unit (MCAU) to perceive the local situation and spatial displacement dynamic features of prediction targets at different scales, attention is ensured on capturing the dynamic information in their spatial dimensions adequately. On this basis, this paper proposes Multi-Scale Spatiotemporal Evolution Attention (MSEA) unit, which further integrates the spatial change features perceived by MCAU units in higher channel dimensions, and learns the spatiotemporal evolution characteristics at different scales, effectively predicting the dynamic characteristics and regularities of targets in sensor information.Through experiments on spatiotemporal prediction standard datasets such as Moving MNIST, video prediction dataset KTH, and Human3.6m, PMSTD-Net demonstrates prediction performance surpassing previous methods. We construct the GPM satellite remote sensing precipitation dataset, demonstrating the network’s advantages in perceiving multi-scale spatiotemporal dynamic changes in remote sensing meteorological data. Finally, through extensive ablation experiments, the performance of each module in PMSTD-Net is thoroughly validated.

## 1. Introduction

In recent years, with the rapid development of sensor technology, a large amount of high-precision sensor image data and satellite remote sensing data have been continuously acquired and applied across various societal domains. In the processing and application of visual sensor images captured by sensors and remote sensing meteorological data obtained from satellites, predicting future state changes based on historical sensor data has emerged as a significant research area. During the temporal evolution of sensor image data and remote sensing meteorological data, changes in targets often exhibit more significant dynamic characteristics relative to their background information and are highly stochastic. Current methods exhibit limited accuracy in predicting changes in targets in both temporal and spatial dimensions within sensor image data and satellite remote sensing meteorological data, lacking research into the dynamic characteristics of targets across multiple temporal and spatial scales. Effectively utilizing data acquired from image sensors and satellites to achieve more precise predictions of future state changes has thus become a critical challenge. With the advancement of computing power, AI-based prediction methods are gradually being applied to the processing and application of sensor data. Compared to traditional methods, AI-based approaches can capture high-dimensional feature representations in visual sensor images in an end-to-end manner, extracting change-related characteristics from data in a self-supervised manner without the need for manually designed features. This enables better utilization of sensor information [1]. However, some current deep learning-based methods have not effectively captured and predicted the spatiotemporal movement characteristics of targets.

In the specific spatiotemporal evolution process, we observe the existence of two distinct forms of movement variation. One form involves the localized changes in the predicted target’s state, which occur locally and vary in magnitude, following changes in the predicted target. For instance, this could include the swinging of a person’s arms or the magnitude of leg movement during walking, as well as variations in rainfall amounts in localized regions. The other form entails dynamic changes in the spatial displacement of the predicted target, where the predicted target’s movement range in space can vary greatly. Such changes occur alongside the completion of the predicted target’s motion process, such as the displacement generated during a person’s walking or running, or the migration of rainfall ranges in spatial areas. These two types of changes cannot be accurately predicted using fixed-scale information. Therefore, this paper proposes a predictive network, PMSTD-Net, based on perceiving multi-scale spatiotemporal dynamic changes. Introducing an attention mechanism [2] enables the PMSTD-Net model to adaptively adjust the attention weights of the model according to the dynamic spatiotemporal changes in the predicted target, thereby learning the local situational changes and spatial displacement dynamic evolution patterns of the predicted target [3].

In response to the changing characteristics of two specific forms of movement during spatiotemporal variations, we propose MCAU and MSEA in PMSTD-Net. For capturing spatial features, MCAU employs Multi-Scale Local Situation Attention (MLSA) units to capture detailed features of varying sizes within the predicted target’s local state. Another component of MCAU, the Multi-Scale Spatial Displacement Attention (MSDA) units, utilizes a structure involving large convolutional kernels and dilated convolutions with different dilation rates to perceive movement information across different spatial scales. Following the acquisition of multi-scale spatial motion features, MSEA integrates spatial features in high dimensions and learns their spatiotemporal change characteristics at different scales to predict spatiotemporal state distributions. The network design of PMSTD-Net targets the changing characteristics of local situations and spatial displacements, aligning well with our understanding of spatiotemporal patterns. PMSTD-Net demonstrates excellent predictive capabilities for forecasting changes in human body movements within sensor image data and abrupt changes in local meteorological elements within remote sensing meteorological data. It also effectively predicts changes in human body position activities in sensor images and spatial movement patterns of meteorological data on a larger scale. From the perspective of sensor images, PMSTD-Net accurately predicts future changes in human movements within sensor data, which is applicable, for instance, in autonomous driving for predicting pedestrian movements and enabling more reasoned driving decisions. Similarly, from the perspective of remote sensing meteorology, it forecasts future large-scale movements of precipitation based on obtained remote sensing precipitation data, including local extreme precipitation events. In summary, this research holds significant implications for future issues such as traffic safety in autonomous vehicles and the early prevention of disasters caused by precipitation. Experimental results on four datasets containing sensor image and remote sensing meteorological data demonstrate that PMSTD-Net outperforms recent models in the field of spatiotemporal prediction across all four datasets. The contributions of this paper are as follows:

1. This paper proposes the PMSTD-Net deep learning architecture, composed of Encoder–Translator–Decoder. The Translator, through the Metaformer structure, combines MCAU and MSEA, enabling the study of spatiotemporal variations in prediction targets at different scales. This presents a novel approach to address the stochastic nature of spatiotemporal motion variations in prediction targets.
2. Sensor images and remote sensing meteorological data exhibit complex spatiotemporal variation characteristics. We have designed a novel MCAU and MSEA to address this complexity. The MCAU captures local situation variation features and spatial displacement dynamics of the predicted targets at different scales. Meanwhile, the MSEA unit integrates spatial features across scales and learns their spatiotemporal evolution characteristics.
3. To validate the prediction performance of PMSTD-Net, we evaluated its effectiveness across multiple datasets: Moving MNIST [4], KTH [5], Human3.6 [6], and a precipitation dataset constructed from actual GPM [7] satellite remote sensing images. PMSTD-Net demonstrated state-of-the-art predictive performance on all these datasets. Extensive ablation experiments were conducted to verify the predictive capabilities of each module within PMSTD-Net.

## 2. Related Works

This section focuses on the development of deep learning based spatiotemporal prediction methods and their applications on sensed data.

### 2.1. The Development of Spatiotemporal Prediction Methods

The spatiotemporal prediction task can be specifically represented as follows [8]: Given a spatiotemporal sequence $X_{t}\in R^{w\times h\times c}$ with n historical frames $X=(X_{t-n},\ldots ,X_{t-1},X_{t})$, where t, w, h, and c represent time, width, height, and the number of channels, respectively, the task is to predict future states $Y=(Y_{t},Y_{t+1}\ldots ,Y_{t+m})$ by inputting the n historical frames $X=(X_{t-n},\ldots ,X_{t-1},X_{t})$. Currently, there are many methods based on deep learning to address spatiotemporal prediction tasks, which can be roughly categorized into spatiotemporal prediction methods based on recurrent neural networks, convolutional neural networks, and Vision Transformers.

Recurrent neural networks (RNNs) [9] are widely applied to spatiotemporal prediction tasks due to their inherent recurrent structure. In 2015, ConvLSTM [10] extended FC-LSTM by replacing fully connected layers with convolutional operations, enhancing its ability to capture spatial features. In 2017, PredRNN [11] introduced a novel Spatiotemporal Attention (ST-LSTM) unit on top of ConvLSTM, enabling the simultaneous extraction of temporal and spatial information across LSTM units. Addressing gradient vanishing during training, PredRNN++ [12] (2018) integrated Gradient Highway Units (GHUs) and Causal LSTM structures to capture long-term dependencies. E3D-LSTM [13] (2018) combined 3D convolution and RNNs for spatiotemporal sequence prediction. MIM [14] (2019) separated temporal information into stationary and non-stationary components. MAU [15] (2021) proposed a motion-aware unit to widen the temporal receptive field of prediction units for reliable inter-frame motion information. MotionRNN [16] (2021) decomposed motion into transient changes and motion trends, employing MotionGRU units to analyze spatiotemporal evolution characteristics of targets. SwinLSTM [17] (2023) integrated SwinTransformer with LSTM modules, replacing ConvLSTM’s convolutional structure with an attention mechanism extension. However, error accumulation issues can arise in recurrent neural networks due to their cyclic nature.

Compared to recurrent neural networks, convolution-based neural network designs are relatively simpler. In 2016, DVF [18] introduced a deep voxel flow method that extracts moving pixels from all pixel values to learn the synthesis of video frames. In 2018, PredCNN [19] simulated the dependency between the next frame and video input, designing a cascade multiplication unit (CMU). In 2022, SimVP [8] proposed a video prediction model based on fully convolutional neural networks. Within the encoding–translating–decoding structure, the intermediate translation module relies solely on Inception modules and group convolutions to learn spatiotemporal correlations in sequences. In 2023, MMVP [20] introduced an end-to-end dual-stream video prediction framework, separating motion and appearance information by constructing motion matrices independent of appearance. Building upon SimVP, in 2023, TAU [21] introduced a parallelizable attention module that decomposes temporal attention into intra-frame static attention and inter-frame dynamic attention. They proposed a novel differential divergence regularization method to analyze inter-frame differences. Convolutional neural networks have limitations in capturing temporal evolution characteristics, unable to consider the complex spatiotemporal variations in moving objects in spatiotemporal prediction tasks, and lacking targeted analysis of spatiotemporal motion changes.

### 2.2. The Applications on Sensed Data

Temporal–spatial prediction methods rely heavily on specific real-world applications, playing crucial roles in video prediction and forecasting related variables in remote sensing meteorology. The Human3.6m [6] and KTH [5] datasets for human pose prediction are publicly available video prediction datasets composed of posture and behavioral actions captured by visual sensors of experimental subjects indoors or outdoors, extensively applied in temporal–spatial prediction applications. The KTH dataset comprises images of six actions (walking, jogging, running, boxing, hand waving, and hand clapping) captured by visual sensors, performed by 25 experimental subjects across four different scenarios, playing a significant role in action recognition and behavior prediction from sensor images. The Human3.6m dataset, a human pose prediction dataset, encompasses a total of 3.6 million 3D human poses and corresponding images, including actions such as smoking, photographing, and walking performed by 11 experimental subjects across seven scenarios, predicting different action poses of various individuals in videos. For instance, STMP-Net [22] proposes a network that integrates temporal–spatial memory and motion perception, achieving promising results on the Moving MNIST [4] and KTH datasets. The aforementioned temporal–spatial prediction models like MotionRNN, SwimLSTM, SimVP, and TAU are extensively validated on video prediction datasets to better forecast future state changes based on historical sensor image data.

In the spatiotemporal prediction of remote sensing meteorological variables, precipitation, with its complex spatiotemporal characteristics, has attracted the attention of many researchers for prediction studies. Previous studies have focused on precipitation prediction based on radar sensing data. For instance, MSSTNet [23] proposed an end-to-end multi-scale neural network structure based on 3D convolution, validated on the Moving MNIST dataset and radar echo data. ConvLSTM2D [24] extrapolated radar echoes based on radar precipitation datasets. However, radar observations have limited coverage, and compared to precipitation movements at large scales, satellite remote sensing data offer significant advantages, allowing observation of precipitation changes over large areas from high altitudes. Therefore, the effective utilization of satellite remote sensing data becomes crucial. GPM-IMERG [7], initiated jointly by NASA and JAXA, provides high spatiotemporal resolution precipitation observation data obtained through satellite observation and radar measurements. GPM-IMERG not only offers real-time remote sensing precipitation estimation but also provides historical data and long-term precipitation datasets, facilitating the validation of climate models’ predictive performance. Previously, Pred-SF [25] predicted precipitation by improving the PredRNNv2 [26] structure combined with ERA5 multivariable data and GPM satellite remote sensing precipitation data. However, the structure of recurrent neural networks determines the characteristics of recurrent inputs and outputs, making it challenging to achieve effective parallel processing. Recurrent neural networks cannot learn long-term change patterns based on the overall spatiotemporal sequence input and face issues such as error accumulation during the recurrence process, making it difficult to handle long-term dependencies.

## 3. Method

**Figure 1:**
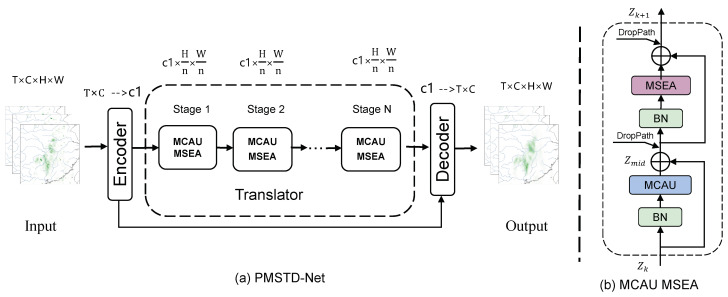
(**a**) The overall architecture of the perceiving multi-scale spatiotemporal dynamics network (PMSTD-Net) consists primarily of the Encoder, Translator, and Decoder modules. (**b**) The structure of the MACU and MSEA modules.

This paper proposes a multi-scale motion change attention network named PMSTD-Net, structured around Encoder, Translator, and Decoder components. As illustrated in Figure 1, historical spatiotemporal sequence data enters PMSTD-Net. Initially, the Encoder integrates the time dimension into the channel dimension to extract high-dimensional features. Subsequently, these high-dimensional features are fed into the Translator, which incorporates Multi-Scale Motion Change Attention Units (MCAU) and Multi-Scale Spatiotemporal Evolution Attention (MSEA) units under the Metaformer structure. Metaforme [27], derived from Transformer, is depicted in detail in Figure 1b, comprising two normalization processing modules and two specific modules. This architecture replaces Transformer’s attention modules with simple spatial pooling operators, demonstrating excellent performance in the field of computer vision, thereby validating its structural design rationale. Therefore, we apply PMSTD-Net to spatiotemporal prediction tasks in sensor image and remote sensing meteorological data, leveraging our proposed Multi-Scale Spatial Attention Unit (MCAU) and Multi-Scale Spatiotemporal Evolution Attention (MSEA) unit. Finally, the learned spatiotemporal state changes are decoded by the Decoder to output predicted future spatiotemporal state images.

### 3.1. Overall Framework

The Encoder mainly consists of stacking n units of (Conv2d+GroupNorm+SiLU). Its function is to perform downsampling and fit nonlinear spatiotemporal features through two-dimensional convolution, group normalization, and the SiLU activation function. This process encodes high-dimensional feature data into low-dimensional features for subsequent learning of spatiotemporal evolution patterns by the Translator module. Specifically, it can be represented as follows:

(1) $$Z_{i+1}=\sigma \left(GroupNorm\left(Conv2d\left(Z_{i}\right)\right)\right),1\leq i\leq m$$

where $Z_{i}$ represents the input to the *i* layer of the Encoder, $Z_{i+1}$ represents the output of the *i* layer of the Encoder, *m* denotes the number of layers in the Encoder, and σ stands for the SiLU activation function.

The Decoder primarily consists of stacking n units of (Conv2D+PixelShuffle+GroupNorm+SiLU) layers. Its role involves upsampling through convolution, pixel shuffling, group normalization, and activation functions, aiming to preserve and restore the temporal and spatial transformation patterns learned by the Translator, thereby recovering the details lost during downsampling. Specifically, it can be represented as follows:

(2) $$Z_{j+1}=\sigma (GroupNorm\left(PixelShuffle\left(Conv2d\left(Z_{j}\right)\right)\right),1\leq j\leq 2m+n$$

where $Z_{j}$ represents the input to the *j* layer of the Encoder, $Z_{j+1}$ represents the output of the *i* layer of the Decoder, *m* denotes the number of layers in the Decoder, and σ stands for the SiLU activation function.

The Translator module primarily accepts low-dimensional features extracted by the Encoder, learning their spatiotemporal evolution patterns. It is composed mainly of Multi-Scale Motion Space Change Attention Unit (MCAU) to capture spatial features and Multi-Scale Spatiotemporal Evolution Attention units to learn spatiotemporal evolution characteristics. These are connected together through the structure of Metaformer to form the MCAU-MSEA block, represented by the following equation:

(3) $$Z_{mid}=Z_{k}+MCAU\left(Norm\left(Z_{k}\right)\right),n\leq k\leq m+n$$

(4) $$Z_{k+1}=Z_{mid}+MSEA\left(Norm\left(Z_{mid}\right)\right),n\leq k\leq m+n$$

where $Z_{k}$ represents the input to the *k* layer of the Translator, $Z_{j+1}$ represents the output of the *k* layer of the Decoder, *m* denotes the number of layers in the Translator, Norm is BatchNorm2d.

As shown in Figure 1, integrating DropPath into the Metaformer structure allows for the random dropping of certain network branches to regularize the network. This prevents overfitting that may arise from increasing the number of intermediate Translator layers. Additionally, it enhances the network’s generalization ability. We employed multiple scales to capture spatiotemporal change features. Depending on the utilization of features at different scales, we reduced the complexity of the model by pruning certain network branches. This adaptation facilitates handling various tasks and improves the stability of network training.

### 3.2. Multi-Scale Spatial Motion Change Attention Unit (MCAU)

In spatiotemporal prediction tasks, the motion changes in the prediction target itself often occur more dramatically compared to changes in its background information. Considering that the motion of spatiotemporal prediction targets involves characteristics of changes at different scales, we proposes the Multi-Scale Motion Change Attention Unit (MCAU). It consists of two modules: the Multi-Scale Spatial Displacement Attention (MSDA) module and the Multi-Scale Local-State Attention (MLSA) module.

To address dynamic spatial displacement, we propose the MSDA. Considering the broad range of position changes in the prediction target across the entire space, we first employ a 7 × 7 large convolutional kernel to capture extensive spatial information. Subsequently, we utilize dilated convolutions with varying dilation rates to enhance the model’s spatial positional awareness at different scales. Previous studies have demonstrated the effectiveness of using larger convolutional kernels for capturing long-term dependencies [28] in models, which is advantageous in spatiotemporal prediction tasks. Spatial position changes often occur throughout the entire spatiotemporal motion process, and large convolutional kernels play a crucial role in accurately predicting these changes by capturing informative features. By employing dilated convolutions with different dilation rates, the receptive field of MSDA is expanded several times over, allowing observations of position feature changes at different scales. This enriches MSDA’s ability to acquire position information, thereby facilitating a more comprehensive capture of features related to large-scale positional movements. Furthermore, we utilize depth-wise separable convolutions to improve computational efficiency. Finally, the GELU activation function is employed to fit nonlinear positional displacement features.

**Figure 2:**
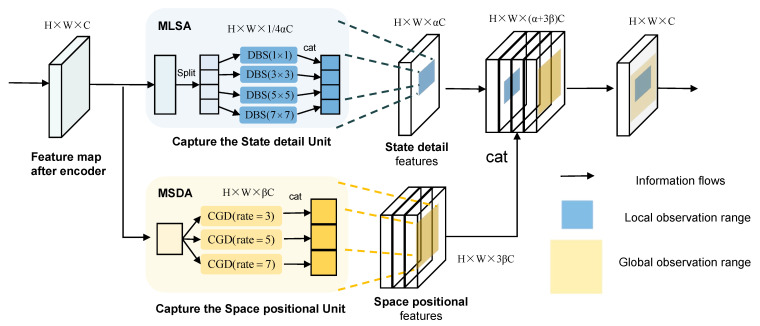
MCAU module main framework and MSDA and MLSA capture spatial displacements and local-state features.

As shown in Figure 2, MSDA extends the feature maps after the Encoder to βC channels. Each time, the features of C channels are captured using CGD modules with different dilation rates to capture their spatial positional features, as indicated by the orange range in Figure 2. The specific formula is represented as follows:

(5) $$CGD_{m}:Y_{a}=\sigma_{1}(Norm(DilaDWConv2d_{7\times 7}\left(X,rate=b\right),a=1,2,3;b=3,5,7$$

(6) $$Y=cat(Y_{1},Y_{2},Y_{3})$$

where *X* represents the input to MSDA, *Y* represents the output of MSDA, and Norm denotes BatchNorm2d. $DilaDWConv2d_{7\times 7}$ with a convolutional kernel size of 7 × 7, where c represents the dilation rate and $\sigma_{1}$ denotes the GELU activation function.

To address the local situation changes, we propose a Multi-Scale Local Situation Attention (MLSA) module. By designing the DBS structure with different scales, smaller convolutions with convolution kernels of 1, 3, 5, 7 are used to capture the small-scale detailed deformation features, and the MLSA pays more attention to the detailed deformation features of the predicted target in the process of spatiotemporal motion changes. Finally, the local fine nonlinear change features are fitted by SiLU activation function. As shown in Figure 2, MLSA, on the other hand, divides the feature map into 1/4αC channels, and for each 1/4αC channel, the *DBS* module is carried out with different-sized convolution kernels to capture its local detailed state features, which reduces the computational resources. The specific form is shown in the blue range of Figure 2, and the specific formula is expressed as follows:

(7) $${DBS}_{n}:Y_{c}=\sigma_{2}(Norm(DWConv2d_{n\times n}\left(X_{c}\right),c=1,2,3,4;n=1,3,5,7$$

(8) $$Y=cat(Y_{1},Y_{2},Y_{3},Y_{4}))$$

where *X* represents the input to MLSA, *Y* represents the output of MLSA, Norm denotes BatchNorm2d, and $\sigma_{2}$ represents the SiLU activation function, while n denotes the size of the convolutional kernel.

MLSA and MSDA capture spatial displacement dynamics and local situation detail information through dilated convolutions with different scales of large and small kernels. They aggregate the captured information of different types and restore the channel number from (α+3β)C to the original number of channels C through 1 × 1 convolutions. The MLSA module and MSDA module are interconnected in terms of their receptive fields, enabling a more detailed analysis of the motion state changes in the predicted targets.

**Figure 3:**
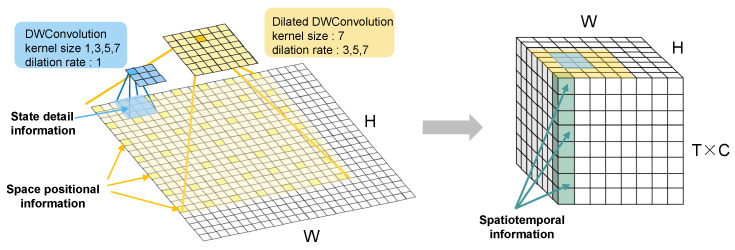
Capture of local situation and spatial displacement information in space as well as fusion of spatial information and learning of its temporal and spatial patterns of change.

As shown in Figure 3, the MSDA module captures the spatial location information through the expansion convolution with different expansion rates of the large convolution kernel [29], which mainly adopts the depth-separable convolution with the convolution kernel of 7, and the expansion rates of 3, 5, and 7. Among them, we can observe that the MSDA module enlarges the sensory field of the space many times through the expansion convolution with different scales, and the expanded convolution information can capture the location information in the whole feature map. The MLSA module captures the detail information through a number of small kernel convolutions. By adopting the depth-separable convolutions with convolution kernels of 1, 3, 5 and 7, respectively, the MLSA traverses the whole feature map and captures the detail information that cannot be concerned by the MSDA, and at the same time adopts the convolution kernels of different scales, which will also make the local detail information more complete and rich. Finally, the learned spatial information features are superimposed according to the time dimension, as shown in the right panel of Figure 3, at which the spatial displacement information and the local attitude information are superimposed together; they are fused by the Multi-Scale Spatiotemporal Evolutionary Attention unit(MSEA) in high dimensions and analyzed for their spatiotemporal evolution characteristics at different scales.

**Figure 4:**
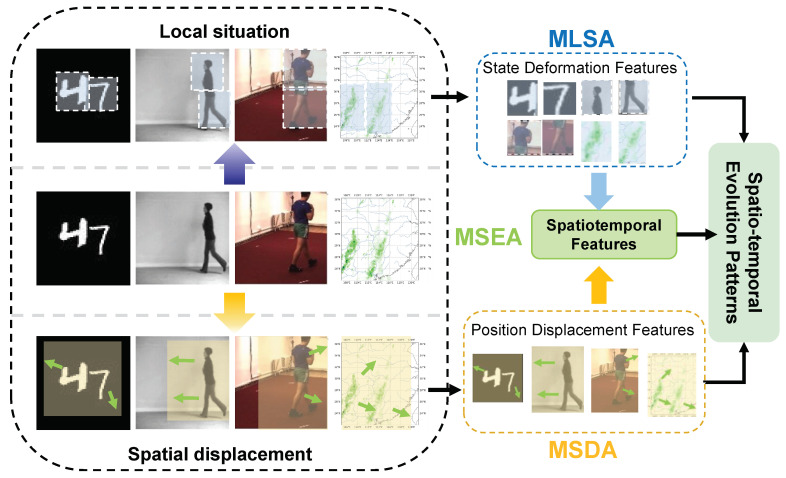
The two existent forms of dynamic changes in the predicted targets on the publicly available dataset Moving MNIST, the video prediction dataset KTH, Human3.6m, and the GPM satellite remote sensing precipitation dataset are captured by MLSA and MSDA in PMSTD-Net at different scales to characterise the localised change in the posture of the predicted targets and the dynamic features of the spatial displacements, respectively, and MSEA is used at different scales to fuse the spatial features and learn their spatiotemporal evolutionary properties.The green small arrows in the figure indicate the trend in the spatial location of the predicted targets.

As shown in Figure 4, PMSTD-Net demonstrates its capability in capturing local-state fine details and spatial displacement features from Moving MNIST, KTH, Human3.6, and GPM satellite remote sensing precipitation datasets. Subsequently, the variations in these features are learned by the MLSA and MSDA modules. These learned features are then input into the MSEA module to integrate the spatial characteristics and learn their temporal evolution patterns, ultimately deriving the spatiotemporal change patterns.

### 3.3. Multi-Scale Spatiotemporal Evolutionary Attention (MSEA) Unit

**Figure 5:**
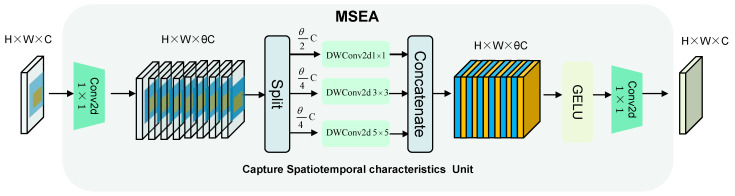
The main framework and structure of MSEA.

In spatiotemporal prediction tasks, spatial characteristics exhibit complex temporal variations, making it challenging to accurately predict the temporal evolution of the target. Therefore, as illustrated in Figure 5, we propose a Multi-Scale Spatiotemporal Evolutionary Attention (MSEA) unit, which builds upon captured local situation change features and spatial displacement dynamic features. In high dimensions, MSEA integrates spatial features captured at different scales and learns spatiotemporal evolution patterns at different scales. The specific formula is as follows:

(9) $$X_{\gamma 1},X_{\gamma 2},X_{\gamma 3}=Split\left(Conv2d_{1\times 1}\left(P_{l}\right)\right)$$

(10) $$Y_{\gamma 1,2,3}=DWConv2d_{n\times n}\left(X_{\gamma 1,2,3}\right),n=1,3,5$$

(11) $$P_{k}=Conv2d_{1\times 1}(GELU(cat\left(Y_{\gamma 1},Y_{\gamma 2},Y_{\gamma 3}\right)$$

where $P_{l}$ represents the input of the MSEA unit and $P_{k}$ represents the output of the MSEA module. In PMSTD-Net, the temporal dimension is fused into the channel dimension. This is achieved by expanding the channel (C) to a higher dimension θC using 1 × 1 convolutional operations. Within this higher dimension, the θC channel dimension is partitioned, and parallel depth-wise separable convolutions are employed at various scales to capture the spatiotemporal evolution patterns. The resulting feature maps are then aggregated, and the GELU activation function is applied to model the nonlinear characteristics of spatiotemporal changes. Finally, the channel count is restored to its original quantity using 1 × 1 convolutional operations.

## 4. Experiments

In this section, PMSTD-Net is experimented on multiple datasets to validate its outstanding performance in spatiotemporal prediction using sensor images and remote sensing meteorological data. Firstly, quantitative and qualitative analyses are conducted on standard spatiotemporal prediction datasets including MovingMNIST, video prediction datasets Human3.6m and KTH. PMSTD-Net demonstrates exceptional performance in predicting randomly moving digits in the standard spatiotemporal prediction datasets, as well as human poses and actions in video prediction datasets. It surpasses previous state-of-the-art results on these datasets across multiple metrics evaluating prediction against truth. Additionally, we construct the GPM satellite remote sensing precipitation dataset and validate PMSTD-Net’s strong competitiveness in predicting the spatiotemporal distribution of actual precipitation on this dataset. Furthermore, ablation experiments are conducted on PMSTD-Net’s proposed modules: the Local Situation Awareness module (MLSA), Spatial Displacement Attention module (MSDA), and Spatiotemporal Evolution Attention module (MSEA) across multiple datasets. These experiments further analyze the performance of multi-scale and individual modules. We replace the three modules with multi-scale Inception modules and single-scale MLSA, MSDA, and MSEA to control model parameters (Param) and floating-point operations (FLOPs). Results demonstrate that PMSTD-Net maintains superior prediction performance with the highest training efficiency.

### 4.1. Dataset Introduction and Experimental Parameter Settings

Below are introductions for the four datasets used in this study: Moving MNIST, KTH, Human3.6m and the GPM precipitation dataset.

The Moving MNIST dataset is the most commonly used dataset in the field of spatiotemporal prediction. Almost all spatiotemporal sequence prediction methods validate their effectiveness on this dataset. The spatiotemporal sequences in the Moving MNIST dataset consist of sequences of 20-frame images, where the first 10 frames predict the subsequent 10 frames. These sequences depict two randomly generated handwritten digits moving within a grid. The initial positions and velocities of the digits are random, and the digits rebound upon touching the edges of the grid, potentially overlapping if they occupy the same position. Theoretically, this random motion of digits can generate countless sequence combinations, significantly increasing the difficulty of spatiotemporal prediction tasks. Such a stochastic generation of predictive images aligns more closely with real-life variations caused by random factors, making it crucial for spatiotemporal prediction research.

The KTH dataset is widely used for validating spatiotemporal prediction tasks. It consists of sensory visual images capturing human actions from visual sensors. The dataset primarily includes six actions (walking, jogging, running, boxing, hand waving, and hand clapping), performed by 25 subjects across four different scenarios. Training involves subjects 1 to 16, while testing uses subjects 17 to 25. Each image is adjusted to predict 20 frames of human actions based on 10 input frames of human motion images. The prediction time step is twice the input time step, placing higher demands on the model’s predictive flexibility. Accurate prediction of future human actions is crucial, particularly in the realm of autonomous driving for real-world traffic scenarios.

The Human3.6m dataset is a collection focused on human pose prediction, comprising sensory visual images of human poses captured by visual sensors. The dataset contains a total of 3.6 million 3D human poses along with corresponding images. It includes actions such as smoking, photographing, and walking performed by 11 experimental subjects across seven scenarios. We primarily utilize the “walking” scenario for predicting human poses. The original image data, sized at 1024 × 1024, is resized to 128 × 128 format. Model training is conducted using segments S1, S5, S6, S7, and S8 of the dataset, while segments S9 and S11 are reserved for model testing. Predictions for the next four images are made based on the first four frames of human walking actions. The Human3.6m dataset enables more effective prediction of human behavioral poses in sensory data.

**Figure 6:**
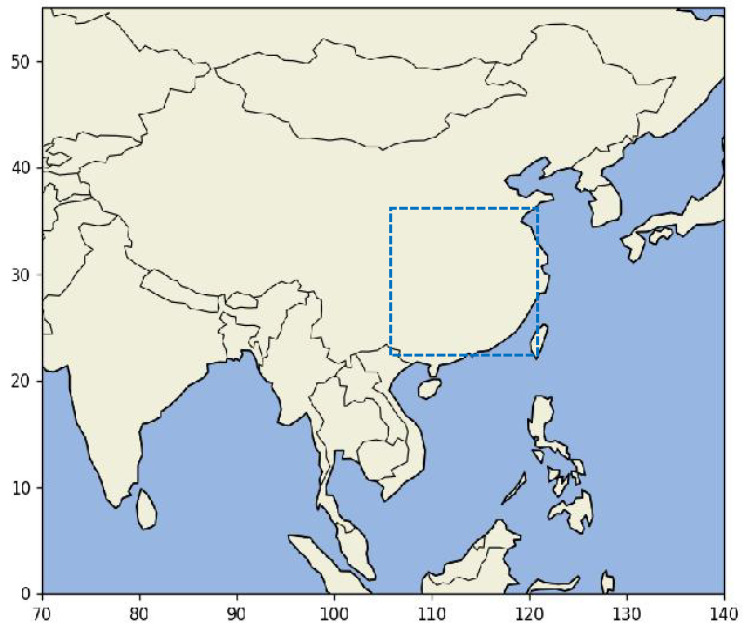
The spatial extent within the blue dotted line is the area included in the GPM satellite precipitation dataset.

We utilized the IMERG precipitation product from the GPM satellite to study the distribution of precipitation in both temporal and spatial dimensions. The GPM satellite remote sensing precipitation dataset has a spatial resolution of 0.1° and a temporal resolution of 30 min. The selected study area primarily covers the southern region of China, ranging from 107° E to 121.3° E in longitude and 22° N to 36.3° N in latitude. This area was chosen due to its frequent susceptibility to flood disasters during the monsoon season, making research into the spatiotemporal distribution of precipitation crucial for natural disaster prevention and mitigation. The time span of the GPM precipitation dataset ranges from April to September each year, covering the years 2017 to 2022, with data from 2017 to 2021 used as the training set and 2022 data used as the test set. The area of the precipitation range we studied is shown in Figure 6.

The training samples $N_{train}$ and testing samples $N_{test}$ for the four datasets, Moving MNIST, KTH, Human3.6m, and the GPM satellite remote sensing precipitation dataset, are presented in Table 1. Here, T and T’ denote the length of the time series for input and prediction data, C represents the number of channels in the input data, and H and W, respectively, indicate the height and width of the input data.

Table 2 presents the training parameters and model settings of PMSTD-Net across four datasets. For Moving MNIST, KTH, and Human3.6m, publicly available datasets, to ensure consistency and avoid potential performance discrepancies due to different experimental conditions, we adopted the experimental setups as reported in the respective benchmark papers. This approach was chosen to maintain experimental fairness in our comparative analysis. Regarding our constructed GPM satellite remote sensing precipitation dataset, PMSTD-Net and the comparison models were evaluated under identical experimental conditions for forecasting precipitation in both temporal and spatial dimensions.

### 4.2. Experiments on Moving MNIST

In the experiments on the Moving MNIST dataset, we compared 15 models: ConvLSTM [10], FRNN [30], PredRNN [11], PredRNN++ [12], MIM [14], LMC [31], E3D-LSTM [13], MAU [15], MotionRNN [16], PhyDNet [32], SimVP [8], Crevnet [33], TAU [21], MMVP [20], and SwimLSTM [17]. These models represent the most outstanding performers in spatiotemporal prediction in recent years. We conducted experiments under the same experimental settings as these models. Evaluation was performed using the same metrics as previous studies: Mean Squared Error (MSE), Mean Absolute Error (MAE), and Structural Similarity Index (SSIM), to assess the effectiveness of predictions.

MSE represents the overall Mean Squared Error between the predicted images and truth, with the specific formula as follows:

(12) $$MSE=\frac{\sum_{i=1}^{n}{\left(x_{i}-y_{i}\right)}^{2}}{n}$$

*MAE* represents the overall Mean Absolute Error between the predicted images and ground truth, with the specific formula as follows:

(13) $$MAE=\frac{\sum_{i=1}^{n}\left|x_{i}-y_{i}\right|}{n}$$

where n represents the number of pixels in the image, $x_{i}$ denotes the truth value, and $y_{i}$ represents the predicted value. The lower the *MSE* and *MAE*, the better the prediction performance.

*SSIM* represents the similarity between the predicted image and the ground truth image, with a maximum value of 1. The specific formula is as follows:

(14) $$SSIM=\frac{\left(2\mu_{x}\mu_{y}+c_{1}\right)\left(2\sigma_{xy}+c_{2}\right)}{\left(\mu_{x}^{2}+\mu_{y}^{2}+c_{1}\right)\left(\sigma_{x}^{2}+\sigma_{y}^{2}+c_{2}\right)}$$

where $\mu_{x}$ is the mean value of x and $\mu_{y}$ is the mean value of y. $\sigma_{x}^{2}$ is the variance of x, $\sigma_{y}^{2}$ is the variance of y, and $\sigma_{xy}$ is the covariance of x and y. $c_{1}={\left(k_{1}L\right)}^{2}$ and $c_{2}={\left(k_{2}L\right)}^{2}$ are constants used to maintain stability, $k_{1}=0.01$, $k_{2}=0.02$, and L is the dynamic range of the pixel values. A higher SSIM means better prediction.

To ensure fair comparison on the Moving MNIST dataset and to prevent fluctuations in evaluation metrics caused by variations in comparative models during replication, we employ the best-performing metrics reported in the aforementioned models for comparison. The experimental setup mirrors that of prior papers, utilizing MSE loss for training, a batch size of 16, Adam optimizer, and OneCycle learning rate scheduler with a learning rate of 0.001 over 2000 epochs. The experiments were conducted using PyTorch on a single NVIDIA 3090 GPU.

From Table 3, we can see that PMSTD-Net achieves the best predictive performance on the Moving MNIST dataset. Compared to the SwimLSTM model, which currently performs best on the Moving MNIST dataset, PMSTD-Net reduces MSE from 17.7 to 14.8, a 16.4% improvement, and increases SSIM from 0.962 to 0.968. Moreover, PMSTD-Net decreases MAE from 60.3 to 49.6, a 17.7% improvement compared to the recent TAU model. These experimental results demonstrate that PMSTD-Net outperforms the current best models in predicting Moving MNIST data.

**Figure 7:**
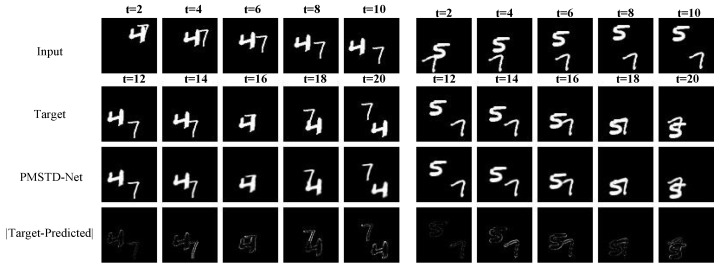
Visualization of the Moving MNIST dataset.

Figure 7 presents the visualized prediction results on the Moving MNIST dataset. The first row displays the ten input frames of digit images, the second row shows the true subsequent ten frames of digit movement, the third row exhibits the predicted subsequent ten frames of digit movement by PMSTD-Net, and the fourth row illustrates the error images between predictions and truths. From the prediction results on the Moving MNIST dataset, it is evident that PMSTD-Net accurately predicts the local motion patterns of overlapping digits ‘4’ and ‘7’, capturing the downward-right movement of ‘4’ and the movement of ‘7’ from directly below to the center, along with changes in their positional shifts. For overlapping digits ‘5’ and ‘7’, PMSTD-Net accurately predicts their initial separation, subsequent overlap, and final separation with only subtle errors at the edges, without inducing blurriness in the overlapping digits. Analysis of the error images between predicted and ground truth movements reveals minimal discrepancies, indicating almost identical alignment with the true movements of the digits. This further underscores the effectiveness of analyzing and studying local motion pattern changes and spatial displacement dynamics during the motion variation process of the target for prediction.

### 4.3. Experiments on KTH

The generalization performance of a model across different datasets is a crucial criterion for evaluating its effectiveness. To assess the generalization performance of the PMSTD-Net model in spatiotemporal prediction tasks, we conducted experiments on the KTH dataset, widely used for validating spatiotemporal prediction tasks.In the prediction experiments on the KTH dataset, we compared nine spatiotemporal sequence models: ConvLSTM [10], DFN [18], MCnet [34], PredRNN [11], PredRNN++ [12], E3D-LSTM [13], MMVP [20], SimVP [8], and TAU [21]. These nine models are widely representative in this dataset. Our model achieved the best prediction performance compared to these models on the KTH dataset. Experimental metrics followed those set in the comparative literature, using the Structural Similarity Index (SSIM) and Peak Signal-to-Noise Ratio (PSNR) to evaluate prediction effectiveness. PSNR, representing the ratio of peak signal to Mean Squared Error (MSE), typically indicates smaller differences between predicted and true images as PSNR values increase. The specific formula for PSNR is as follows:

(15) $$PSNR=20\times \log_{10}\left(\frac{MAX}{\sqrt{MSE}}\right)$$

where *MAX* is the maximum value of pixels in the image and *MSE* stands for Mean Squared Error (*MSE*), the effect of SSIM and PSNR metrics of PMSTD-Net on KTH dataset with other models are shown in Table 4.

**Figure 8:**
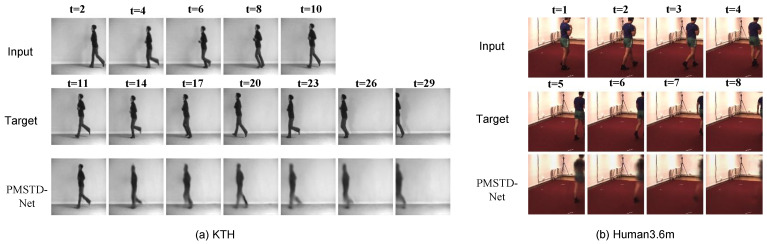
Visualization of the predicted effects of the dataset.

From Table 4, it can be observed that PMSTD-Net demonstrates significantly superior predictive performance on the KTH dataset compared to other models. Compared to the TAU model, which currently exhibits the best predictive performance on the KTH dataset, the SSIM has improved from 0.911 to 0.916, and the PSNR has increased from 34.13 to 34.65. In Figure 8a, we visualize the KTH dataset. By inputting the preceding 10 frames of an individual running and comparing the output of the subsequent 20 frames of predicted images with the ground truth, we can observe that the overall action posture, positional transition from the right side of the room to the left side, and the extent of movement are accurately predicted. The dynamic spatial displacement features of the human body are well-predicted. Regarding local details, such as the ‘lifting leg’, ‘placing leg’, and ‘arm swinging’ actions, although there may be slight blurring at the edges during the prediction process, the overall action postures are accurately predicted, resulting in excellent predictive performance.

### 4.4. Experiments on Human3.6m

We conducted predictive experiments on the Human3.6m dataset, comparing models including ConvLSTM, PredRNN, PredRNN++, MIM, E3D-LSTM, MotionRNN, PhyDNet, and SimVP—eight spatiotemporal prediction models extensively evaluated on the Human3.6m dataset. Following the experimental setup of comparative models, we evaluated the Human3.6m dataset using Mean Squared Error (MSE), Mean Absolute Error (MAE), and Structural Similarity Index (SSIM). Experimental results are presented in Table 5.

Table 5 illustrates that PMSTD-Net outperforms other models significantly in terms of prediction accuracy on the Human3.6m dataset. The Mean Squared Error (MSE) decreased from 316 to 302 compared to the previously best-performing SimVP, representing a 4.4% improvement. Similarly, the Mean Absolute Error (MAE) decreased from 1510 to 1350 compared to SimVP, indicating a 10.6% enhancement. Moreover, the Structural Similarity Index (SSIM) increased from 0.904 to 0.910 compared to SimVP. Our model achieves the best prediction performance across the Human3.6m dataset. Figure 8b provides visualizations of human pose prediction on the Human3.6 dataset, accurately predicting both the movement and positions of the moving poses. Compared to the background information captured within the room, the variations in human body movements are more pronounced. PMSTD-Net effectively captures the dynamic nature of position changes as the human gradually disappears in the image, demonstrating its capability in predicting positional changes effectively.

### 4.5. Experiments on GPM Satellite Remote Sensing Precipitation

We constructed the GPM satellite remote sensing precipitation dataset to evaluate the spatiotemporal distribution prediction performance of PMSTD-Net. We selected PredRNN++, PredRNNv2, MotionRNN, SimVP, and TAU models, which have demonstrated outstanding performance in spatiotemporal prediction over the past five years, as comparative models for experimentation. The GPM satellite remote sensing precipitation data accumulates precipitation every 30 min, with generally low precipitation amounts. Therefore, we categorized precipitation into three levels: 0.1 mm/30 min, 1 mm/30 min, and above 5 mm/30 min, representing no precipitation, light precipitation, and moderate to heavy precipitation, respectively. We studied the spatiotemporal distribution by inputting historical data of precipitation distribution over three hours (6 frames) to predict the future three hours (6 frames). Considering the practical significance of precipitation forecasting, we analyzed the prediction effectiveness of precipitation spatiotemporal distribution using meteorological metrics, primarily Mean Squared Error (MSE), Critical Success Index (CSI), and Precision. The formulas for CSI and Precision are given as follows:

(16) $$CSI=\frac{TP}{TP+FP+FN}$$

(17) $$Precision=\frac{TP}{TP+FP}$$

where *N* represents the number of samples, *TP* denotes the count of pixels where both predicted and ground truth values indicate precipitation, *FP* represents the count of pixels where precipitation is predicted but not observed in ground truth, and *FN* signifies the count of pixels where precipitation is not predicted but observed in ground truth. Here, *CSI* indicates the proportion of correctly predicted precipitation areas to the actual observed precipitation areas, while Precision represents the proportion of correctly predicted precipitation areas to the total predicted precipitation areas. A lower MSE suggests better overall prediction performance, while higher values of CSI and Precision indicate greater accuracy in predicting precipitation regions. The forecasted precipitation indicator data is presented in Table 6.

In Table 6, we observe that the PMSTD-Net model surpasses previous methods in terms of MSE, CSI 0.1, CSI 1, CSI 5, Precision 0.1, and Precision 5 metrics. The Precision 1.0 metric is slightly inferior to the top-performing TAU model, but PMSTD-Net outperforms TAU significantly in the other five metrics. Specifically, compared to TAU, PMSTD-Net shows a 2.8% improvement in CSI 0.1, a 3.7% improvement in CSI 5, a 5.3% improvement in Precision 0.1, a 4.1% improvement in Precision 5, and a 4.5% improvement in MSE. These results clearly demonstrate the outstanding performance of our model in predicting precipitation from GPM satellite remote sensing data.

**Figure 9:**
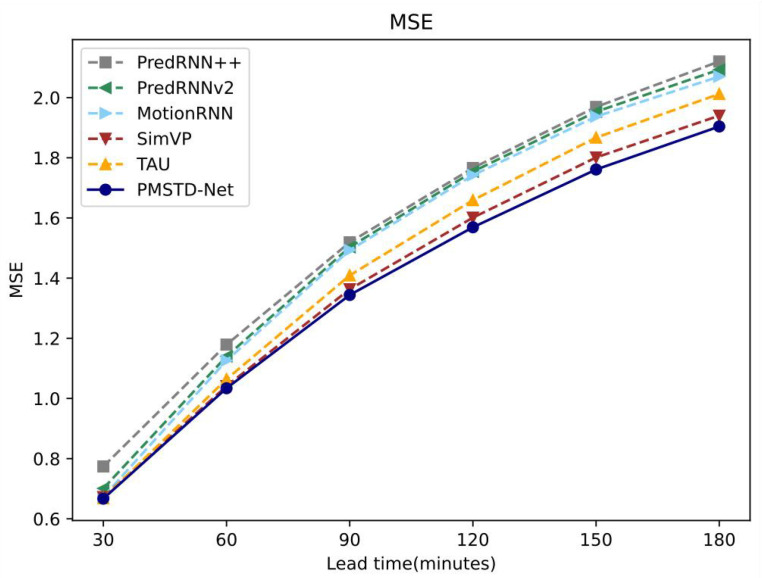
Variation of MSE with forecast duration for different modelling models on GPM satellite remote sensing precipitation dataset.

Figure 9 illustrates the variation of MSE with increasing forecast duration for PMSTD-Net and other models. Overall, as the forecast duration increases, the MSE of each model rises, indicating a degradation in forecasting performance with longer prediction times in precipitation forecasting. Remarkably, PMSTD-Net consistently maintains the lowest MSE among all models, outperforming the other five models. This underscores the superior performance of the PMSTD-Net model in precipitation prediction.

**Figure 10:**
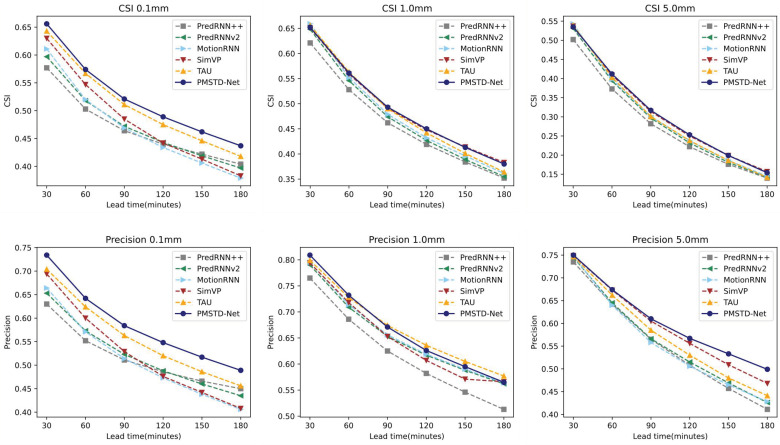
Changes in CSI and Precision metrics with increasing forecast length for different models on the GPM satellite precipitation dataset.

Figure 10 displays the trends of CSI and Precision metrics of PMSTD-Net and other models as the forecast duration increases. CSI and Precision are analyzed with precipitation thresholds of 0.1 mm/30 min, 1 mm/30 min, and 5 mm/30 min, respectively. It is evident that as the forecast duration increases, PMSTD-Net achieves the best performance across all three different threshold values of CSI. While for Precision, the forecast performance for the 1 mm/30 min precipitation threshold is slightly lower than TAU, the performance for the other two precipitation thresholds improves compared to other models as the forecast duration increases.

**Figure 11:**
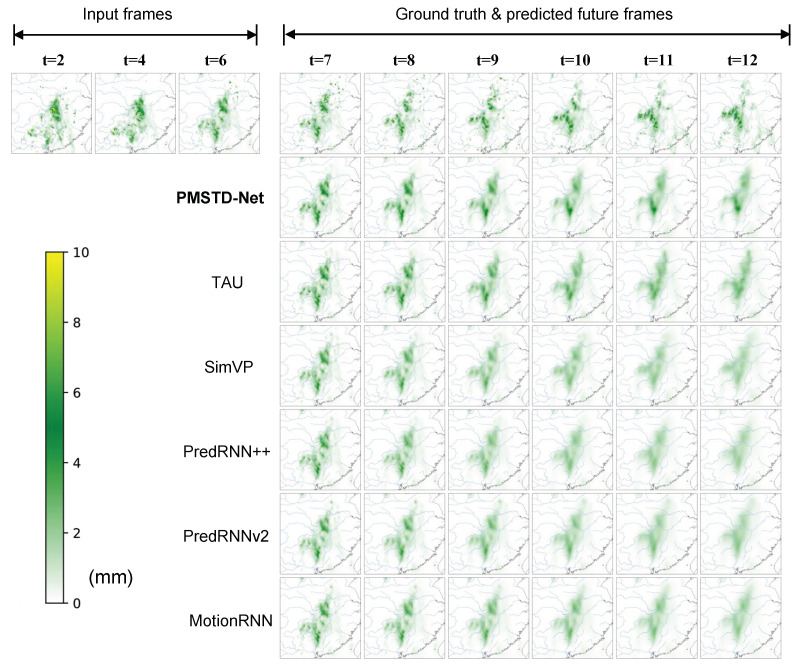
Visualization of the predictive effects of different models in the GPM satellite remote sensing precipitation dataset.

Figure 11 visualizes the spatiotemporal distribution of predicted precipitation from GPM satellite remote sensing data by PMSTD-Net and other models. The first row on the left shows the spatiotemporal distribution sequence of the input preceding 6 frames of precipitation, while the first row on the right represents the actual subsequent 6 frames of precipitation distribution. Below are the predicted results of PMSTD-Net and various comparative models. From the figure, it can be observed that PMSTD-Net exhibits superior overall performance in precipitation prediction. It performs better in forecasting small precipitation areas and local areas with relatively large precipitation amounts during the forecasting process compared to other models. PMSTD-Net can predict the movement range of precipitation areas and abrupt changes in precipitation amounts in local areas effectively. In contrast, other models tend to predict a decrease in precipitation amounts over time for this region and fail to predict an increase in precipitation amounts. This suggests that our attention to dynamic changes in spatial displacement and local situational changes in spatiotemporal prediction is effective.

### 4.6. Ablation Experiments

#### 4.6.1. Ablation Experiments with Deletion or Addition of Modular Forms

In order to validate the performance of PMSTD-Net and the effectiveness of the proposed motion change decomposition, we add or delete three modules of PMSTD-Net: the MLSA module, the MSDA module, and the MSEA module, respectively, and conduct experiments on the Moving MNIST, Human3.6m, and GPM precipitation datasets.

In this section, four sets of experiments are set up: Method 1 contains only the MLSA module; Method 2 contains only the MSDA module; Method 3 contains both the MLSA module and the MSDA module; and Method 4 is the PMSTD-Net containing these three modules.The performance of each module is verified through the form of control variables. In the experiment, MSE loss is uniformly used to train the loss function; Batchsize is set to 16; Adam is set as the optimiser; OneCyle learning rate regulator is set; the learning rate is 0.001; the number of training rounds for the Moving MNIST, Human3.6m, and GPM satellite precipitation datasets are 100, 100, and 30 epoch; and the experiment was performed on a single NVIDIA 3090 GPU using Pytorch.

According to the ablation experiments conducted on the Moving MNIST, Human3.6m, and GPM satellite remote sensing precipitation datasets, Method 3, which integrates MLSA and MSDA, demonstrates superior performance compared to Method 1 and Method 2, as shown in Table 7. This suggests that spatial motion decomposition into spatial positional changes and local-state changes significantly enhances prediction performance. When the MSEA module is added to Method 3 to form PMSTD-Net, it generally outperforms Method 3 in most experimental metrics. However, in Precision 5 of the GPM satellite precipitation dataset, the performance is not as good as Method 1 and Method 2. This might be attributed to insufficient learning of the characteristics of changes in some local areas with high precipitation. In our future work, we will focus on researching this aspect in more detail.

**Figure 12:**
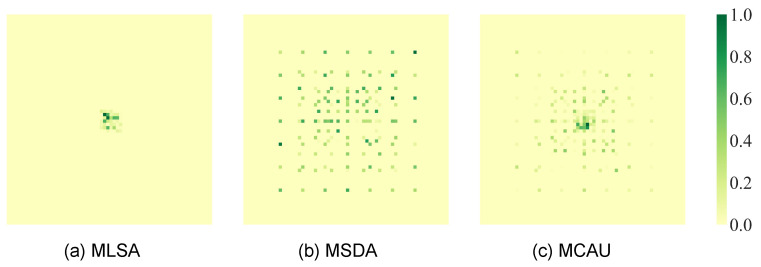
The receptive field effect diagrams of the single-layer MLSA module, MSDA module, and their combination, the MCAU module, on the first ten frames of Moving MNIST.

Following the article’s visualization method of receptive fields, we visualized the receptive fields of the single-layer MLSA, MSDA, and their combination, MCAU, on the Moving MNIST dataset. As shown in Figure 12, the receptive field of the single-layer MLSA module is concentrated only in the central region, focusing excessively on detailed features and thus unable to predict spatial positional changes in the target. In contrast, the receptive field of the MSDA module is more evenly distributed across the entire image, but due to its broad receptive state, it fails to capture detailed features of the prediction target. Combining the MLSA and MSDA modules, however, enables better capture of both the prediction target itself and the surrounding areas with larger-scale motion position changes.

#### 4.6.2. Ablation Experiments under the Replacement Module

Considering that adding or removing modules may affect the overall model’s parameter count and computational efficiency, thus potentially biasing the ablation experiment results, this study further extends the ablation experiments regarding the performance of each module in PMSTD-Net. We expand the evaluation from three aspects: model parameter count, floating-point operation count, and training time per epoch. These are compared with the effects of single-scale MLSA (Local-State Attention units), single-scale MSDA (Spatial Position Attention units), and single-scale (MSEA) (Spatiotemporal Evolution Attention units) used in SimVP, along with the Inception module. This ensures a fair comparison of the effectiveness of each module under similar parameter counts and computational resources, confirming the efficacy of multi-scale effects. Since the Moving MNIST dataset significantly influences model performance perception, the ablation experiments are primarily conducted on this dataset. Model performance is evaluated using MSE, MAE, and SSIM metrics. Additionally, considering the trade-off between model prediction accuracy and computational efficiency, we analyze the parameter count (Params), floating-point operation count (FLOP), and training time for each ablation experiment.

The experiments were divided into five main comparison groups and PMSTD-Net, where Method 1 contains two base $Inception_{\left(3,5,7,11\right)}s$ and a single-scale $MSEA_{\left(3\right)}$ module, Method 2 replaces one base $Inception_{\left(3,5,7,11\right)}$ with an MLSA module based on Method 1, Method 3 is a combination of a single-scale $MLSA_{3\times 3}$, $MSDA_{\left(3\right)}$ employing a convolutional kernel of 3 and an expansion rate of 3, Method 4 employs a combination of MSDA, MLSA and a single-scale $MSEA_{\left(3\right)}$ combination, and Method 5 adopts a combination of base $Inception_{\left(3,5,7,11\right)}$, MLSA and MSEA.

**Figure 13:**
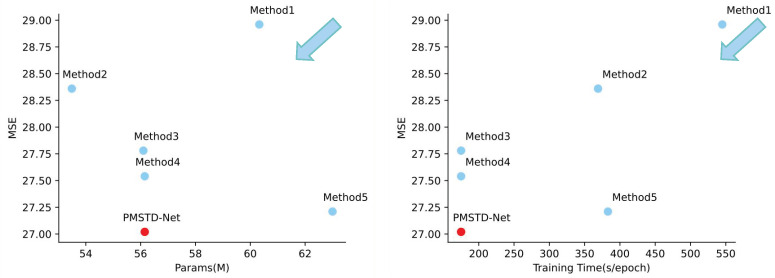
Demonstration of the effect of PMSTD-Net and each experimental group on Params and Training Time for MSE on the Moving MNIST dataset, where PMSTD-Net is shown in red and the comparison experiments are shown in blue. Arrows indicate the direction of model optimisation.

Table 8 and Figure 13 show that compared to Method 1, PMSTD-Net does not necessarily perform better solely by adopting a multi-scale approach, indicating ineffective learning of multi-scale information in both spatial and temporal domains. Although Method 2 has fewer parameters than PMSTD-Net, its longer training time compared to PMSTD-Net demonstrates the performance of the MSDA module. The comparison between Method 3 and PMSTD-Net proves the necessity of capturing multi-scale spatiotemporal information. Method 4 highlights the importance of the multi-scale MSEA module compared to PMSTD-Net. In the comparison between Method 5 and PMSTD-Net, despite the large description amount and high computational resources, Method 5’s performance is not as good as PMSTD-Net, showcasing the superior performance of the MLSA model. Overall, all experimental groups, with relatively small differences in experimental parameters and computational load, show that PMSTD-Net achieves the best prediction results in the shortest training time. This also demonstrates the significance of PMSTD-Net in studying motion changes in spatiotemporal prediction. The designed MLSA, MSDA, and MSEA structures exhibit excellent effects in predicting the local situation changes and spatial displacements of the prediction targets in spatiotemporal prediction, providing strong support for the study of predicting complex movements in spatiotemporal prediction tasks.

## 5. Discussion and Conclusions

In sensing images and remote sensing meteorological data, the prediction targets exhibit complex spatiotemporal variability, which manifests more pronounced dynamic characteristics relative to their background information. We have developed a neural prediction network named PMSTD-Net, which is based on perceptual multi-scale spatiotemporal dynamic changes. It captures the dynamic variations in local trends and spatial displacement features of prediction targets at different scales during spatiotemporal prediction processes, thereby learning their future spatiotemporal distribution patterns. In our study, PMSTD-Net has achieved more precise predictions of future spatiotemporal distribution states for remote sensing meteorological data and sensor image data. This advancement is crucial for mitigating natural disasters such as floods caused by uneven spatiotemporal precipitation distributions, and for predicting future pedestrian movements to enhance traffic safety.

PMSTD-Net is structured into three main components: an Encode–Translator–Decoder framework. The Encoder module encodes high-dimensional sensory image and remote sensing meteorological data into low-dimensional feature space, facilitating subsequent learning by the Translator module on spatiotemporal evolution patterns. The Translator module consists of Multi-Scale Spatial Motion Change Attention Units (MCAUs) and Multi-Scale Spatiotemporal Evolution Attention (MSEA) units under the Metaformer framework. MCAU comprises Multi-Scale Local-State Attention (MLSA) units and Multi-Scale Spatial Displacement Attention (MSDA) units. MLSA captures local detail variations in sensing images and remote sensing meteorological data across different scales, while MSDA captures spatial displacement characteristics over large spatial extents. Subsequently, MSEA integrates these features to learn their temporal evolution patterns. Finally, the Decoder module decodes the spatiotemporal evolution patterns learned by Translator back to the original dimensions, yielding future spatiotemporal state distributions.

In three publicly available standard spatiotemporal prediction datasets, Moving MNIST, KTH, and Human3.6m, we achieved the best prediction performance. Specifically, compared to previous best results on the Moving MNIST dataset, PMSTD-Net showed a 16.4% improvement in Mean Squared Error (MSE) and a 17.7% improvement in Mean Absolute Error (MAE), with the structural similarity index increasing from 0.962 to 0.968. On the KTH dataset, PMSTD-Net improved upon the previous best prediction performance, with the structural similarity index (SSIM) increasing from 0.911 to 0.916 and the Peak Signal-to-Noise Ratio (PSNR) increasing from 34.13 to 34.65. On the Human3.6m dataset, compared to the previous best prediction performance, PMSTD-Net achieved a 4.4% improvement in MSE, a 10.6% improvement in MAE, and an increase in SSIM from 0.904 to 0.910. Subsequently, we constructed the GPM satellite dataset and validated PMSTD-Net’s best forecasting performance on GPM satellite precipitation data by comparing it with spatiotemporal prediction models from the past five years. We also visualized the changes in various indicators of the precipitation model as the forecast length increased. PMSTD-Net demonstrates significant competitiveness in spatiotemporal precipitation forecasting.

Ablation experiments on multiple datasets were conducted to validate the spatiotemporal prediction performance of each module and the combination of the Local Situation Attention (MLSA) module, Spatial Displacement Attention (MSDA) module, and Temporal Evolution Attention (MSEA) module proposed in PMSTD-Net. We also purposely replace the MLSA, MSDA, and MSEA modules with multi-scale Inception modules and single-scale MLSA, MSDA, and MSEA modules to control the number of model parameters (Param) and the number of floating-point operations (FLOPs); PMSTD-Net still achieves the best prediction results with the highest training efficiency.

In addition to using remote sensing imagery for spatiotemporal prediction, there are many other important applications in the field of remote sensing image processing. Particularly in complex scenarios, effective utilization of specific types of sensor images can achieve various complex tasks. For instance, tracking and detecting ships using satellite remote sensing images can assist in monitoring maritime activities and enhancing maritime safety management [35]. Long-term monitoring and prediction of ocean temperatures are crucial for climate change research and marine resource management, helping scientists and policymakers understand the dynamic changes in marine ecosystems [36]. Overall, the applications of remote sensing image processing across various domains demonstrate its significant potential and value in addressing complex real-world problems. We hope our work contributes to more accurate predictions of sensor image information and remote sensing meteorological data, thereby providing assistance for future advancements in artificial intelligence in handling sensor images and remote sensing meteorological data.

## Figures and Tables

**Table 1:** Dataset statistics.

	$N_{\mathrm{train}}$	$N_{\mathrm{test}}$	(C,T,H,W)	(C,T’,H,W)
Moving MNIST [4]	10,000	10,000	(1,10,64,64)	(1,10,64,64)
KTH [5]	5200	3167	(1,10,128,128)	(1,20,128,128)
Human3.6m [6]	2624	1135	(3,4,128,128)	(3,4,128,128)
GPM Precipitation	7280	1456	(1,6,144,144)	(1,6,144,144)

**Table 2:** Setting of training parameters and model parameters on the four datasets.

Parameter Type	Moving MNIST	KTH	Huamn3.6m	GPM Precipitation
Seed	1	1	1	1
Batchsize	16	4	16	16
Training epochs	2000	100	100	30
Learning rate	0.001	0.001	0.01	0.001
Layers of Encoder	4	2	2	2
Layers of Decoder	4	2	2	2
Layers of Translator	8	7	5	5
Number of Encoder channels	64	64	64	64
Number of Decoder channels	64	64	64	64
Number of Translator channels	512	256	256	256

**Table 3:** Comparison of PMSTD-Net with models from recent years on the Moving MNIST dataset on MSE, MAE, and SSIM metrics, where (↓) lower or (↑) higher indicates better predictions.

Method	Conference	MSE (↓)	MAE (↓)	SSIM (↑)
ConvLSTM [10]	NIPS 2015	103.3	182.9	0.707
FRNN [30]	CVPR 2017	69.7	-	0.813
PredRNN [11]	NIPS 2017	56.8	126.1	0.867
PredRNN++ [12]	ICML 2018	46.5	106.8	0.898
MIM [14]	CVPR 2019	44.2	101.1	0.910
LMC [31]	CVPR 2021	41.5	-	0.924
E3D-LSTM [13]	ICLR 2018	41.3	87.2	0.910
MAU [15]	NIPS 2021	27.6	-	0.937
MotionRNN [16]	CVPR 2021	25.1	-	0.920
PhyDNet [32]	CVPR 2020	24.4	70.3	0.947
SimVP [8]	CVPR 2022	23.8	68.9	0.948
Crevnet [33]	ICLR 2020	22.3	-	0.949
MMVP [20]	ICCV 2023	22.2	-	0.952
TAU [21]	CVPR 2023	19.8	60.3	0.957
SwimLSTM [17]	ICCV 2023	17.7	-	0.962
PMSTD-Net	-	**14.8**	**49.6**	**0.968**

**Table 4:** Comparison of SSIM and PSNR metrics under different model outputs on KTH, where (↓) lower or (↑) higher indicates better predictions.

Method	SSIM (↑)	PSNR (↑)
ConvLSTM [10]	0.712	23.58
DFN [18]	0.794	27.26
MCnet [34]	0.804	25.95
PredRNN [11]	0.839	27.55
PredRNN++ [12]	0.865	28.47
E3D-LSTM [13]	0.870	29.31
MMVP [20]	0.906	27.54
SimVP [8]	0.905	33.72
TAU [21]	0.911	34.13
PMSTD-Net	**0.916**	**34.65**

**Table 5:** Comparison of MAE, MSE and SSIM metrics under different model outputs on Human3.6m, where (↓) lower or (↑) higher indicates better predictions.

Method	MSE/10 (↓)	MAE/100 (↓)	SSIM (↑)
ConvLSTM [10]	50.4	18.9	0.776
PredRNN [11]	48.4	18.9	0.781
PredRNN++ [12]	45.8	17.2	0.851
MIM [14]	42.9	17.8	0.790
E3D-LSTM [13]	46.4	16.6	0.869
MotionRNN [16]	34.2	14.8	0.846
PhyDNet [32]	36.9	16.2	0.901
SimVP [8]	31.6	15.1	0.904
PMSTD-Net	**30.2**	**13.5**	**0.910**

**Table 6:** Comparison of MSE, CSI and Precision Metrics of Different Models on GPM Satellite Remote Sensing Dataset, where (↓) lower or (↑) higher indicates better predictions.

Method	MSE (↓)	CSI 0.1 mm (↑)	CSI 1.0 mm (↑)	CSI 5.0 mm (↑)	Precision 0.1 mm (↑)	Precision 1.0 mm (↑)	Precision 5.0 mm (↑)
PredRNN++ [12]	1.555	0.463	0.455	0.281	0.511	0.618	0.578
PredRNNv2 [26]	1.524	0.467	0.469	0.295	0.514	0.656	0.587
MotionRNN [26]	1.507	0.459	0.475	0.300	0.499	0.659	0.583
SimVP [8]	1.403	0.470	0.485	0.310	0.508	0.645	0.617
TAU [21]	1.447	0.501	0.482	0.301	0.548	**0.673**	0.604
PMSTD-Net	**1.382**	**0.515**	**0.486**	**0.312**	**0.577**	0.666	**0.629**

**Table 7:** PMSTD-Net correlation metrics for MLSA, MSDA, and MSEA modular ablation experiments on Moving MNIST, Human3.6m, and GPM satellite precipitation datasets, where (↓) lower or (↑) higher indicates better predictions.

Dataset	Type/Index	Method 1	Method 2	Method 3	PMSTD-Net
–	MLSA	√		√	√
	MSDA		√	√	√
	MSEA				√
Moving MNIST	MSE (↓)	39.05	34.81	32.21	**27.02**
	MAE (↓)	105.51	96.54	89.91	**78.08**
	SSIM (↑)	0.907	0.919	0.926	**0.939**
Human3.6m	MSE/10 (↓)	38.5	31.5	30.8	**30.2**
	MAE/100 (↓)	16.2	13.6	15.3	**13.5**
	SSIM (↑)	0.895	0.907	0.905	**0.910**
GPM Precipitation	MSE (↓)	1.463	1.576	1.487	**1.382**
	CSI 0.1 mm (↑)	0.448	0.269	0.467	**0.515**
	CSI 1.0 mm (↑)	0.474	0.436	0.463	**0.486**
	CSI 5.0 mm (↑)	0.262	0.222	0.268	**0.312**
	Precision 0.1 mm (↑)	0.484	0.278	0.513	**0.577**
	Precision 1.0 mm (↑)	0.625	0.639	0.638	**0.666**
	Precision 5.0 mm (↑)	**0.663**	0.662	0.630	0.629

**Table 8:** Replacement ablation of the modules of PMSTD-Net on the Moving MNIST dataset, where $Inception_{\left(3,5,7,11\right)}$ consists of using convolutional kernels of 3, 5, 7, 9, and the $MLSA_{3\times 3}$ and $MSDA_{\left(3\right)}$ consist of 3 convolutional kernels and an inflated convolution with an inflation rate of 3, where (↓) lower or (↑) higher indicates better predictions.

–	Type/Index	Method 1	Method 2	Method 3	Method 4	Method 5	PMSTD-Net
Part A	$Inception_{\left(3,5,7,11\right)}$	√				√	
	$MLSA_{3\times 3}$			√			
	MLSA		√		√		√
Part B	$Inception_{\left(3,5,7,11\right)}$	√	√				
	$MSDA_{\left(3\right)}$			√			
	MSDA				√	√	√
Part C	$MSEA_{\left(3\right)}$	√			√		
	MSEA		√	√		√	√
Moving MNIST	MSE (↓)	28.96	28.36	27.78	27.54	27.21	**27.02**
	MAE (↓)	81.74	81.06	79.57	79.12	78.47	**78.08**
	SSIM (↑)	0.935	0.936	0.938	0.938	**0.939**	**0.939**
	Params (↓)	60.33 M	**53.49 M**	56.10 M	56.15 M	63.10 M	56.15 M
	FLOPs (↓)	20.04 G	**18.29 G**	18.96 G	18.97 G	20.73 G	18.97 G
	Training Time (↓)	545 s	369 s	**175 s**	**175 s**	383 s	**175 s**

## Data Availability

Data are contained within the article.
